# Detecting Novel Ototoxins and Potentiation of Ototoxicity by Disease Settings

**DOI:** 10.3389/fneur.2021.725566

**Published:** 2021-08-17

**Authors:** Allison B. Coffin, Robert Boney, Jordan Hill, Cong Tian, Peter S. Steyger

**Affiliations:** ^1^Washington State University Vancouver, Vancouver, WA, United States; ^2^Rewire Neuro, Portland, OR, United States; ^3^Department of Biomedical Sciences, School of Medicine, Creighton University, Omaha, NE, United States; ^4^National Center for Rehabilitative Auditory Research, Portland, OR, United States

**Keywords:** screening, novel ototoxins, COVID-19, *in silico*, *in vivo*, *in vitro*, cell lines, zebrafish

## Abstract

Over 100 drugs and chemicals are associated with permanent hearing loss, tinnitus, and vestibular deficits, collectively known as ototoxicity. The ototoxic potential of drugs is rarely assessed in pre-clinical drug development or during clinical trials, so this debilitating side-effect is often discovered as patients begin to report hearing loss. Furthermore, drug-induced ototoxicity in adults, and particularly in elderly patients, may go unrecognized due to hearing loss from a variety of etiologies because of a lack of baseline assessments immediately prior to novel therapeutic treatment. During the current pandemic, there is an intense effort to identify new drugs or repurpose FDA-approved drugs to treat COVID-19. Several potential COVID-19 therapeutics are known ototoxins, including chloroquine (CQ) and lopinavir-ritonavir, demonstrating the necessity to identify ototoxic potential in existing and novel medicines. Furthermore, several factors are emerging as potentiators of ototoxicity, such as inflammation (a hallmark of COVID-19), genetic polymorphisms, and ototoxic synergy with co-therapeutics, increasing the necessity to evaluate a drug's potential to induce ototoxicity under varying conditions. Here, we review the potential of COVID-19 therapies to induce ototoxicity and factors that may compound their ototoxic effects. We then discuss two models for rapidly detecting the potential for ototoxicity: mammalian auditory cell lines and the larval zebrafish lateral line. These models offer considerable value for pre-clinical drug development, including development of COVID-19 therapies. Finally, we show the validity of *in silico* screening for ototoxic potential using a computational model that compares structural similarity of compounds of interest with a database of known ototoxins and non-ototoxins. Preclinical screening at *in silico, in vitro*, and *in vivo* levels can provide an earlier indication of the potential for ototoxicity and identify the subset of candidate therapeutics for treating COVID-19 that need to be monitored for ototoxicity as for other widely-used clinical therapeutics, like aminoglycosides and cisplatin.

## Introduction

Drug-induced ototoxicity gained widespread recognition in the 1940's due to the discovery of hearing loss in patients receiving the then-novel aminoglycoside antibiotic streptomycin (1). Since then, ototoxicity is increasingly recognized as an adverse outcome for a variety of drug classes including other antimicrobial compounds (e.g., erythromycin, vancomycin), platinum-based chemotherapy agents (e.g., cisplatin, carboplatin), loop diuretics (e.g., ethacrynic acid, furosemide, bumetanide), and acetyl salicylic acid (1). More than 700 exogenous neurotoxic chemicals and chemical mixtures pose a risk to sensory functions (2–4).

Ototoxicity encompasses both cochleotoxicity and vestibulotoxicity. Cochleotoxicity is defined as drug-induced damage to the peripheral auditory system, including cochlear sensory hair cells, neurons, and supporting cells, resulting in hearing loss and/or tinnitus. Similarly, vestibulotoxicity occurs when drugs target the peripheral vestibular sensory cells, neurons and supporting cells, leading to dizziness, vertigo, and loss of balance. This paper will focus more on cochleotoxicity as less is known about the vestibulotoxic effects of many drugs. However, the *in vitro* and *in vivo* models we discuss for identifying emerging ototoxins are likely applicable to both cochleotoxicity and vestibulotoxicity.

In the current pandemic, symptoms such as hearing loss, tinnitus, and dizziness have been reported by patients with COVID-19 (5). Several repurposed drugs that are candidate COVID-19 therapeutics also known to be ototoxic. Therefore, drug-induced ototoxicity is likely prevalent in patients in COVID-19 drug trials, underscoring the need to both evaluate drug ototoxicity before clinical trials and for audiometric monitoring of subjects in clinical trials (6). There are excellent model systems to identify emerging ototoxins before these drugs reach the clinic; it is not ideal if the potential for ototoxicity first arises in patients after the therapeutic has received regulatory approval for widespread clinical use (Phase 4). *In vivo* experiments in mammals are clearly ideal, as the intact mammalian cochlea most closely resembles the human cochlea. However, these are both time- and labor-intensive and best for validating putative ototoxins identified by other means. Alternative biological models provide excellent platforms for rapid identification of putative ototoxins, including cochlear cell lines and the larval zebrafish lateral line. Both models are highly amenable to moderate- to high-throughput screening for ototoxic potential in new drugs or compound libraries, deciphering the molecular mechanisms of toxicity by known ototoxins, and testing protective therapies [e.g., (7–11)]. We review and discuss their advantages in identifying putative ototoxins, including novel drugs and those being repurposed as COVID-19 therapeutics. Both models provide an objective system for screening without *a priori* assumptions about ototoxic potential and for testing multiple drug combinations (12, 13). We then discuss the untapped potential of *in silico* screening using computational models; an approach widely used for toxicity screening in other tissues such as the heart and liver (14–16) but rarely for ototoxin identification (17, 18). We focus on the strategic use of these systems and how researchers can employ these biological and computational models for identifying novel ototoxins.

Moreover, investigation of drug-induced ototoxicity has usually involved healthy preclinical models, without considering the disease setting, e.g., inflammation, co-treatment with other drugs, and genetic susceptibility, in which these drugs are used that can exacerbate ototoxicity. Thus, validating the potential for ototoxicity should also be conducted in disease settings (19, 20). Here, we review ototoxic potential of several COVID-19 therapeutics and discuss factors that may exacerbate ototoxic effects. Zebrafish models are also useful for understanding how inflammation, drug–drug interactions, and other factors may contribute to COVID-19 drug ototoxicity.

## Onset of Cochleotoxicity

Several excellent reviews extensively describe cochleotoxicity, so we only briefly summarize here as a prelude to discussing ototoxic potential of COVID-19 therapeutics (21–23). Cochleotoxicity requires that drugs or their toxic metabolites gain access to cochlear tissues and fluids. Many hydrophilic ototoxic drugs cross the strial blood-labyrinth barrier (BLB) and enter strial tissues before clearing into the endolymph that bathes the apical surfaces of sensory hair cells. The molecular mechanisms by which these drugs cross the BLB remain unknown, and different ototoxic drugs use likely different pathways, e.g., the anti-cancer drug cisplatin likely utilizes OCT2 (organic cation transporter 2) and CTR1 (copper transporter 1) transporters (24, 25); while aminoglycoside antibiotics could utilize TRPV1 (transient receptor potential V1) and TRPV4 channels (26–28). Once in the endolymph, ototoxic drugs typically enter hair cells to induce their cytotoxic effect (29). The predominant pathway for ototoxins to enter hair cells via functional mechanoelectrical transduction (MET)-dependent pathways, including passage through large, non-selective MET channels permeable to organic cations (30–33). Besides killing sensory hair cells, ototoxic drugs can also disrupt cochlear biochemical and metabolic homeostasis to induce hearing loss. Fibrocytes in the lateral wall are involved in multiple processes such as potassium circulation, support of the blood supply, and regulation of cochlear inflammatory responses (34). Accumulation of ototoxic drugs in the stria vascularis can ultimately damage the generation of the endolymphatic potential (EP), leading to reduced auditory sensitivity due to a reduced transduction current (35–37). Ototoxic drugs can also damage spiral ganglion neurons disrupting transmission of the mechanically transduced signals from hair cell to the central auditory system for auditory perception (38, 39). While ototoxicity manifests in several ways, hair cells are often a direct or indirect target.

## Candidate COVID-19 Pharmacotherapeutics With Known Ototoxicity

Viral infections such as measles can cause structural damage to the inner ear and nervous system, leading to hearing loss (40). COVID-19 infections are potentially, yet rarely associated with hearing loss (41–43). However, hearing loss in patients may also occur as adverse events following the use of therapeutics to treat COVID-19 infections. Here, we review several known or suspected ototoxins.

### Chloroquine and Hydroxychloroquine

Chloroquine (CQ) and hydroxychloroquine (HCQ) are quinine-related compounds for treating malaria, systemic lupus erythematosus, and rheumatoid arthritis. Chloroquine also exhibits antiviral properties including inhibiting endosome-mediated viral entry, viral uncoating, and proteolytic processing (44, 45). The repurposing of CQ and HCQ to treat COVID-19 is based on their potential to inhibit SARS-CoV-2 virus replication *in vitro* (46). Common side effects of CQ and HCQ include hearth arrhythmia, liver and kidney toxicity, hypoglycemia, retinopathy, muscle weakness, and ototoxicity including hearing loss, tinnitus, and balance deficits, as well as worsening preexisting hearing loss (47–49). As members of the quinine family, one transient ototoxic side effect of CQ and HCQ is a reversible block of the MET channel (32). Radiolabeled CQ is deposited in the stria vascularis and the planum semilunatum of pigmented, but not albino, rats (50), potentially damaging the melanin-containing cells in the stria vascularis, with secondary lesions in sensory hair cells. However, CQ and HCQ can directly to damage hair cells in the zebrafish lateral line, as discussed below (51). The lack of clinical monitoring for CQ-induced ototoxicity is a concern as CQ dosing in COVID-19 trials is significantly higher than for malaria treatment (52).

### Azithromycin

Azithromycin is a macrolide antibiotic with anti-inflammatory and anti-viral properties and is used in combination with CQ/HCQ to treat COVID-19. Side-effects associated with azithromycin include sensorineural hearing loss, tinnitus, and imbalance (45), with an increased risk of sensorineural hearing loss in patients with chronic obstructive pulmonary disease [COPD; (53)] Oral azithromycin induced reversible reductions in transient evoked otoacoustic emissions (TEOAEs) in guinea pigs (54). Middle ear delivery of azithromycin caused greater damage to the inner hair cells than outer hair cells in a dose dependent manner in the basal region of guinea pig cochleae (24). Studies have shown that azithromycin combined with CQ can speed COVID-19 recovery by reducing viral load (55), yet the combined effects of administering both drugs simultaneously on the hearing of COVID-19 survivors remain unknown.

### Lopinavir-Ritonavir

Lopinavir-ritonavir is an anti-retroviral therapy for human immunodeficiency virus (HIV) type I. This drug combination was repurposed to significantly reduce acute respiratory syndrome and mortality caused by Middle East respiratory syndrome coronavirus (MERS-CoV) or SARS-CoV-1 infections and is therefore of interest for SARS-CoV-2 (56). Although lopinavir-ritonavir may promote recovery in patients hospitalized with severe COVID-19 (57), another study showed no significant benefit when compared with standard of care treatment (58). A case study showed reversible bilateral hearing loss in patients with HIV type I after co-treatment with lopinavir-ritonavir (59), although the mechanism remains unknown.

### Ribavirin

Ribavirin interferes with viral replication by inhibiting viral mRNA synthesis and is used to treat respiratory syncytial virus (RSV) infections, hepatitis C, and some viral hemorrhagic fevers. In COVID-19 patients, triple therapy of ribavirin, lopinavir-ritonavir, and interferon (IFN)-β-1b shortened hospital stays and decreased viral shedding compared to lopinavir-ritonavir therapy alone (60). Treatment with pegylated (PEG)-IFN/ribavirin combination therapy caused severe unilateral or bilateral sudden hearing loss that is reversible after treatment discontinuation in some cases, but is permanent in others (45). There is limited data on the ototoxic effects of ribavirin, and it is possible that IFN/ribavirin ototoxicity is due to non-pegylated IFNs alone rather than ribavirin. However, hearing loss caused by IFN therapy is usually reversible (61), whereas (PEG)-IFN/ribavirin therapy can cause permanent hearing loss (62), suggesting that ribavirin itself may be ototoxic, with a potentiating or synergistic effect where co-treatment with ribavirin exacerbates IFN ototoxicity.

### Remdesivir

Remdesivir, an antiviral, adenosine nucleotide analog drug, is currently the only FDA-approved COVID-19 therapy, with significantly shortened hospital stays and reduced mortality rates (63). Remdesivir inhibits viral replication by binding to RNA-dependent viral polymerase, leading to early completion of RNA transcription (64). There is as yet no direct study of remdesivir ototoxicity, however, other adenosine nucleotide analogs, e.g., Rabavirin, can cause irreversible unilateral or bilateral hearing loss and tinnitus after treatment in patients with chronic hepatitis C (65), suggesting a potential ototoxic side effect for remdesivir.

### Interferons

Interferons (IFNs) are released by numerous cell types to fight invading pathogens, primarily viruses. Type I IFNs (IFN-α and IFN-β) possess robust antiviral and immunomodulatory capabilities and are produced via the TLR7/9-MyD88-interferon regulatory factor (IRF) 7 pathway (66). Interferon therapy for COVID-19 was tested due to the effectiveness of IFN-α and IFN-β against SARS-CoV-1 *in vitro* (67). Recent clinical trials showed that IFN-α therapy significantly reduced viral shedding and levels of inflammatory biomarkers, whereas IFN-β therapy improved virologic clearance; both therapies led to improved recovery in patients with COVID-19 (68, 69). Clinical studies have shown reversible hearing loss in patients receiving either IFN-α or IFN-β therapy (61, 70) via an unknown mechanism, while co-treatment with IFN and other therapies, such as ribavirin can lead to permanent hearing loss (62). Elevated ABR thresholds were observed in albino Swiss mice after treatment with IFN-α2A, likely due to fibroblast cell toxicity in the spiral limbus (71). This study also provided evidence that IFN toxicity may be due to a reversible disturbance of cochlear metabolic homeostasis, rather than structural damage.

### Ivermectin

Ivermectin is a broad-spectrum antiparasitic drug repurposed to treat COVID-19 based on an *in vitro* study showing inhibition of SARS-CoV-2 viral replication (72). Ivermectin is primarily vestibulotoxic, including vertigo and dizziness (45), however, there is very limited knowledge of the underlying mechanism(s).

## Risk Factors That Exacerbate Ototoxicity

Ototoxicity can be exacerbated by other factors such as co-morbidities and synergistic drug interactions. Here we briefly discuss some primary factors that exacerbate ototoxicity.

### Inflammation

Viral and bacterial infections typically induce inflammatory responses that can modulate cochlear uptake of drugs or change their cochlear pharmacokinetics and pharmacodynamics (PK/PD), thereby modulating ototoxicity. Preclinical models with systemic bacteriogenic inflammation (that did not alter drug serum levels) increased cochlear uptake of aminoglycosides and exacerbated the extent of cochleotoxicity (19). More intense inflammatory responses can elevate serum levels of aminoglycosides that presumptively worsen ototoxicity outcomes (19). Inflammation also potentiates cisplatin-induced ototoxicity (73). Permanent hearing loss is observed in patients with multidrug-resistant tuberculosis (MDR-TB) due to aminoglycoside ototoxicity, and individuals with MDR-TB and HIV co-infection have a much higher risk developing aminoglycoside-induced hearing loss compared to individuals without HIV infection (74). Cochlear tissues also participate in the inflammatory response induced by systemic immunogenic stimuli, as well as middle ear or intra-cochlear immunogenic stimuli from bacteria or cellular debris (19, 40, 75, 76). Inflammation enhances cochlear uptake of ototoxins likely through sensitization of drug permeant ion channels (e.g., TRPV1, TRPV4, TRPA1), strial vasodilation, and increased permeability in the blood-strial and blood-perilymph barriers (19, 22, 77). One hallmark of COVID-19 infection is a robust systemic inflammatory response with high cytokine levels, including IFNs, interleukins, and chemokines (78), involved in the initiation, regulation, and resolution of immune response against invading pathogens. Therefore, it is possible that severe systemic inflammation in COVID-19 patients could increase the risk of drug-induced hearing loss when therapeutics used for treating COVID-19 infections are also known or suspected ototoxins.

### Renal Clearance

Renal insufficiency decreases the serum clearance of known ototoxins like aminoglycosides and cisplatin from blood (79, 80), resulting in increased cochlear exposure to circulating ototoxins and therefore increased ototoxic damage. Major causes of renal insufficiency include glomerulonephritis, high blood pressure, or diabetes mellitus. Sepsis and acute inflammation can initiate renal insufficiency and kidney failure (81), exacerbating inner ear exposure to ototoxins. Renal insufficiency also occurs with increasing age, along with the decreased glomerular filtration rate associated with aging (82–85) and can partially account for the increased incidence of aminoglycoside- and cisplatin-induced ototoxicity in older patients. Risk factors for COVID-19 morbidity include increased age and diabetes, suggesting that patients hospitalized for COVID-19 may already have increased risk for ototoxicity due to renal insufficiency.

### Hypoxia, Anoxia, Ischemia

Viral infections can cause hearing loss by diminishing blood supply to the cochlea, thereby reducing oxygen levels. Two major complications in hospitalized COVID-19 patients are low blood oxygen and respiratory distress, also called silent hypoxia. It is suggested that silent hypoxia in patients with COVID-19 is multifactorial: (i) the hypoxia inducible factor (HIF) transcription factor responsible for inducing cellular responses to hypoxia also increases ACE-2 expression that can facilitate entry of the virus into cells; and (ii) hypoxia leads to more severe tissue damage by contributing to the “cytokine storm” and endothelial damage (86). These factors combined lead to severe tissue damage and are a major cause of death resulting from COVID-19. Cochlear hair cells require a sustained oxygen supply due to high metabolic activity and are very sensitive to hypoxia. In addition, disruption of the blood-oxygen supply will have deleterious effects on the stria vascularis activity leading to diminished cochlear potentials and histologic changes (36, 87). SARS-CoV-2 infection is also associated with an increased risk of ischemia caused by blood clots in capillaries, including the capillary beds in the stria vascularis, which may lead to hearing loss. COVID-19 is associated with increased rates of both arterial and venous thromboses in the pulmonary and systemic vasculature. The well-accepted mechanism of thrombosis due to COVID-19 starts with the innate immune system activation to fight against SARS-CoV-2 invasion. Several factors are involved in the activation of the contact pathway of coagulation, including, innate immune cells, platelets, endothelial cells, intravascular tissue factor, and neutrophil release of extracellular traps (88). Thus, a reduced blood/oxygen supply to the inner ear can enhance drug ototoxicity (89).

### Potentiating and Synergistic Effects

Potentiated ototoxicity occurs when a non-ototoxic drug (e.g., pancuronium bromide) or medical condition (e.g., inflammation, see above) is combined with an ototoxin, resulting in increased ototoxicity. Vancomycin, a glycopeptide antibiotic commonly prescribed in neonatal intensive care units (90, 91), can exacerbate aminoglycoside-induced ototoxicity in preclinical models (92). Loop diuretics are co-administered with neuromuscular blocking agents, e.g., pancuronium bromide or vecuronium bromide, for patients requiring respiratory assistance (via intubation and ventilation), which can result in potentiated cochleotoxicity (93, 94). Intubation and ventilation are frequent procedures for patients with COVID-19 admitted to intensive care units. The muscle relaxants required for intubation may also potentiate the known ototoxicity of candidate COVID-19 therapies.

Synergistic ototoxicity occurs when the ototoxic outcome of combined treatment is greater than the sum of ototoxicity via individual treatments. The co-administration of azithromycin and HCQ simultaneously heightens the risk of ototoxicity (95). Loop diuretics are commonly used to reduce high blood pressure and edema and can cause transient hearing loss when administered alone. Loop diuretics block the Na^+^-K^+^-Cl^−^ co-transport of potassium into marginal cells, diminishing the EP (35). By unknown mechanisms, this results in greater entry of aminoglycosides into endolymph, and rapid hair cell death at higher levels than when the aminoglycoside or loop diuretics are delivered alone (96–98). Hospitalized COVID-19 patients often receive several drugs in combination and may be at additional risk for ototoxicity, even if individual drugs are minimally ototoxic in healthy individuals. Auditory cell lines and the zebrafish lateral line are robust platforms for testing drug combinations and studies in cochlear cell lines demonstrate synergistic cytotoxicity of proposed COVID-19 therapies (13), as discussed below.

### Genetic Polymorphisms

Susceptibility to ototoxicity can be enhanced by genomic and mitochondrial polymorphisms. The mitochondrial polymorphism A1555G and C1494T in the mitochondrial gene MTRNR1 encoding the mitochondrial 12S ribosomal subunit greatly exacerbates susceptibility to aminoglycoside-induced ototoxicity (99, 100). Aminoglycoside interaction with mitochondrial 12S ribosomal subunit results in inaccurate translation of mitochondrial proteins, reducing protein synthesis by up to 30–40% and a reduction in cellular respiration, leading to hair cell death and hearing loss (101). A hair cell-specific knockout of genomic *Lmo4*, a transcriptional regulator of cellular apoptosis, enhances susceptibility to cisplatin-induced ototoxicity (102), as do other genetic polymorphisms (103). LMO4 promotes cell survival via JAK/STAT-mediated activation of anti-apoptotic genes (102). It is currently unknown how genetic polymorphisms may potentiate (or attenuate) ototoxicity in COVID-19 patients.

Collectively, there is strong evidence that several candidate therapeutics for COVID-19 in clinical trials are known or potential ototoxins, and that medical conditions commonly observed or co-therapeutics used in COVID-19 patients can exacerbate ototoxicity. Given that most COVID-19 drugs to date offer only modest improvement, such as shortening the hospital stay by a few days, it's important to focus attention on drugs with minimal impact on hearing and vestibular function in those hospitalized for COVID-19. With over 900 drugs in clinical trials for COVID-19 alone, a moderate- or even high-throughput screening of candidate COVID-19 therapeutics will offer an excellent opportunity to determine the ototoxic potential of these drugs to ensure optimal auditory and vestibular outcomes in selecting safe and effective drugs for clinical treatment of COVID-19.

## Models for Ototoxicity Studies

Cochlear cell lines and the zebrafish lateral line offer several advantages in that high-throughput screening is possible, allowing researchers to interrogate a library of compounds to identify potential ototoxins. These models are also amenable to rapid dose- and time-response studies to fully explore the parameter space occupied by a putative ototoxin before embarking on more laborious mammalian studies. We expand on these themes below.

### Cochlear Cell Lines

Many *in vitro* ototoxicity studies employ the HEI-OC1 cell line. These cells, named for their “birthplace”—the House Ear Institute, were derived from the organ of Corti of an embryonic mouse from the H-2Kb-tsA58 (Immortomouse) transgenic line, which allows for conditional immortalization of cells harvested from these animals (8). HEI-OC1 cells, like other cultured cell lines, offer the advantage of relative ease of use and amenability to high-throughput screening. Putative ototoxins can be added to the culture medium and cell density measured with standard cell viability measures such as the MTT assay. One caution, however, is that HEI-OC1 cells are not hair cells; like other cell lines, they proliferate readily (at 33°C, and differentiate over 2 weeks at 39°C), while hair cells are post-mitotic and fully differentiated. Cell viability assays are not sufficient to distinguish between reduced cell viability and reduced cell proliferation. Several studies have combined MTT assays for cell viability with specific cytotoxicity or apoptosis assays (e.g., caspase activation or Annexin-V labeling), leading to isolation of drugs that cause auditory cell death (13, 104).

In 2016, the Kalinec group that created this cell line conducted an exhaustive examination of HEI-OC1 cell responses to several known ototoxins, including acetaminophen, cisplatin, and multiple aminoglycoside antibiotics (7). While several drugs reduced cell viability, including cisplatin, acetaminophen, gentamicin, and neomycin, only cisplatin increased cytotoxicity. Interestingly, both cisplatin and acetaminophen increased caspase 3/7 activation, despite a lack of acetaminophen-induced cytotoxicity. A similar disconnect between caspase activation and cell death was noted in 2012 by Chen et al. in gentamicin-treated HEI-OC1 cells (105). It is unclear why HEI-OC1 cells do not show the expected cytotoxicity from aminoglycoside exposure; Kalinec et al. speculated that surrounding cell types (e.g., supporting cells) are necessary for “normal” ototoxic responses (7). These studies demonstrate that while HEI-OC1 cells are valuable for studies of cisplatin cytotoxicity in the auditory periphery, caution is warranted in extrapolating data interpretation to inner ear hair cells.

HEI-OC1 cells have been widely used to study cellular responses to known ototoxins, particularly cisplatin, and to examine putative protective compounds. For example, dozens of studies show that cisplatin-induced damage to auditory cells is modulated by oxidative stress, inflammation, and autophagy [e.g., (106–111)]. Several compounds effectively protect HEI-OC1 cells from cisplatin toxicity, including antioxidants such as alpha-lipoic acid and ebselen (106, 112, 113). Ebselen has now advanced to clinical trials for cisplatin otoprotection, although results are not yet available (clinicaltrials.gov). Interestingly, the antidiabetic drug metformin also conferred protection from cisplatin toxicity in HEI-OC1 cells (108). This finding was recently confirmed *in vivo* in the zebrafish lateral line and mouse cochlea, demonstrating concordance across multiple model systems (114). While cisplatin is a well-studied ototoxin, these findings collectively demonstrate that HEI-OC1 cells are a tractable system to understand ototoxic responses and test potential therapies.

HEI-OC1 cells are also an excellent test bed for development of less ototoxic variants of essential drugs. A particularly interesting study used HEI-OC1 cells to determine the relative toxicity of platinum-based anti-cancer compounds, with the goal of developing a less toxic version of cisplatin (115). Unfortunately, Monroe et al. (115) found that the monofunctional platinum (II) compounds phenathriplatin and pyriplatin were as cytotoxic to HEI-OC1 cells as cisplatin (115). While these results did not lead to chemotherapy agents with lower ototoxic potential, they highlight the strengths of cochlear cell lines for rapid determination of potential ototoxicity before candidate therapeutics are tested clinically.

A prescient 2014 study used HEI-OC1 cells to screen several HIV retroviral agents and several drugs significantly reduced viability of auditory cells, including efavirenz, ritonavir, delavirdine, nelfinavir, and tenofovir (13). In some cases, therapeutically-relevant combinations of HIV drugs (e.g., Combivir) were more cytotoxic than their individual constituents (AZT and lamivudine, i.e., cytotoxic synergy), raising concerns for hearing loss as a side effect of long-term antiviral therapy in HIV patients. These findings are of greater interest in light of the COVD-19 pandemic. As discussed above, ritonavir-lopinavar, currently in clinical trials for COVID-19, is reported to cause hearing loss in HIV patients, but the mechanism is unknown. In addition, in 2020 remdesivir received emergency use authorization for hospitalized COVID-19 patients, despite modest success in clinical trials (63, 116). While all drugs are needed early in the COVID-19 pandemic, including those with only limited benefit, studies like the one by Thein et al. in 2014 (13) showcase the need to rapidly identify emerging ototoxins to guide selection of COVID-19 therapeutics with minimal auditory side-effects. Cell lines are also an excellent platform for mechanistic studies once ototoxicity is established.

HEI-OC1 cells and cochlear hair cells share many responses to known ototoxins, including up-regulating pro-inflammatory responses. For example, the cytokine TNF-α causes cytotoxicity in HEI-OC1 cells and IFNγ exacerbates this damage (117), suggesting that IFN therapy in COVID-19 patients could potentiate hearing loss due to systemic inflammation in this patient population. Further, the combination of TNF-α and IFNγ increases HEI-OC1 cell sensitivity to cisplatin damage, while reducing inflammatory cytokines attenuates cisplatin cytotoxicity (117–119). We do not yet understand the extent to which COVID-19 therapies, nor the disease itself, modulate cell type-specific inflammation, nor the potential consequences of this inflammation on hearing loss.

The auditory cell lines UB/OC-1 and UB/OC-2 share key features with the HEI-OC1 line. The UB (University of Bristol) lines were derived at embryonic day 13 (E13) from the organ of Corti of the transgenic Immortomouse line (120). Originally created to study cellular development and regeneration, UB/OC cells express hair cell markers including the transcription factor *Brn3c* and important hair bundle proteins *Myo6* and *Myo7a* (120). The UB lines and HEI line show relatively similar sensitivity to cisplatin, with concentrations ~20 μM causing significant loss of cell viability (105, 121). However, UB cells are not frequently used for ototoxicity studies, with only a few studies examining cellular damage and protection mechanisms associated with the known ototoxins cisplatin or gentamicin (121–124). To our knowledge, these cell lines have not been used to identify and characterize novel ototoxins. They may, however, be an untapped resource for high-throughput screens of putative ototoxic compounds.

### Zebrafish Lateral Line

In the last 20 years, the larval zebrafish lateral line has emerged as an excellent model for rapid *in vivo* screening of putative ototoxins, understanding ototoxic mechanisms, and developing protective therapies. The lateral line is an externally-located system of sensory organs (neuromasts) on the head and body of the animal. Like auditory cell lines, lateral line hair cells are exposed to the external medium, making drug incubation and visualization a simple process. Unlike auditory cell lines, however, each neuromast contains hair cells and supporting cells and each hair cell receives both afferent and efferent innervation. Therefore, the lateral line combines the convenience of a cell line with the power of an *in vivo* preparation for sensory perception assays.

**Figure 1:**
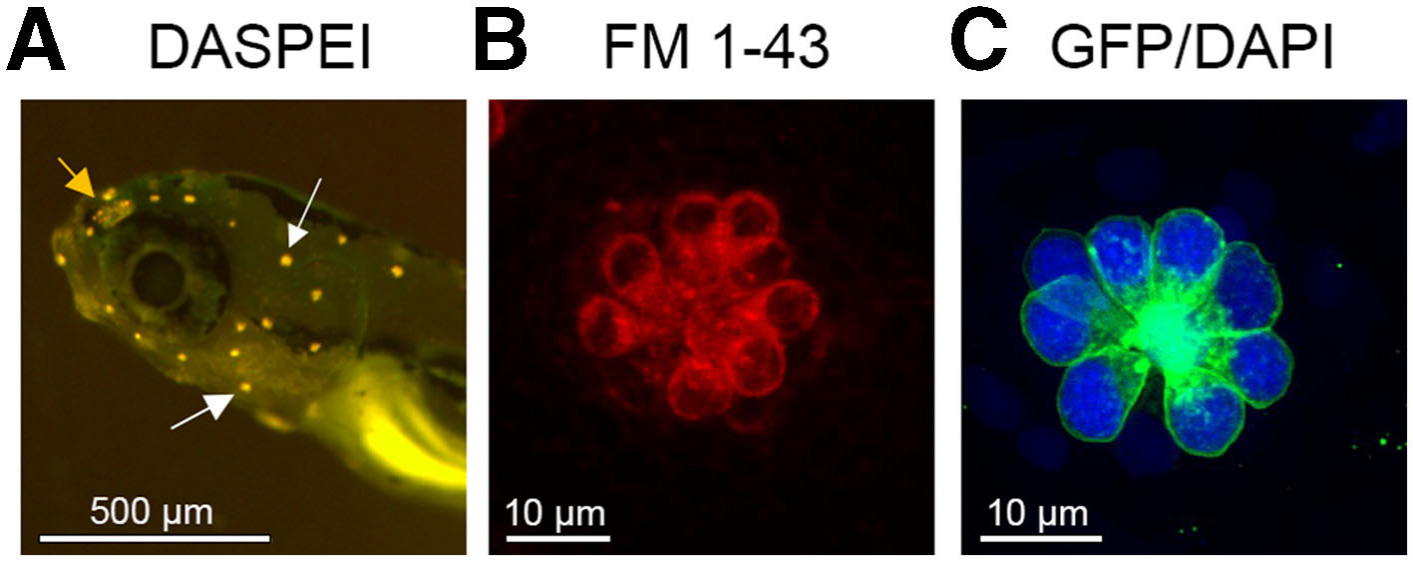
Lateral line visualization using fluorescent markers. **(A)** Larval zebrafish head showing labeling with the vital dye DASPEI. White arrows denote examples of labeled neuromasts. The orange arrow shows the olfactory epithelium, which also labels with DASPEI. **(B)** A single neuromast labeled with the vital dye FM 1–43, that enters hair cells through the MET channel. **(C)** A single neuromast from a Brn3c:mGFP transgenic zebrafish, where hair cells express membrane-bound GFP (green). This fish was also live incubated with DAPI (blue) to selectively label hair cell nuclei.

Lateral line damage is most often analyzed using fluorescence assays. Vital dyes such as DASPEI and FM1-43 are rapidly and specifically taken up by lateral line hair cells and can be visualized with fluorescent microscopy (125–127). DASPEI is a mitochondrial potential dye commonly used for a rapid, holistic assessment of hair cell health, while FM1-43 or fluorescently-tagged aminoglycosides are taken up through MET channels at the apical surface of the hair bundle and therefore serve as a proxy for MET channel function (11, 128–130). Other vital dyes include Yo-Pro-1 and DAPI; generally considered nuclear labels for fixed tissue, both dyes are specifically taken up by lateral line hair cells in an intact animal (127, 131). Often, screens for potential ototoxins use a combination of dyes, allowing for simultaneous visualization of multiple hair cell compartments (9, 132). Figure 1 illustrates common methods for labeling lateral line hair cells described in this paragraph.

In addition to vital dyes, several transgenic lines express hair cell-specific fluorescent proteins, allowing for rapid hair cell assessment. For example, the *Tg*(Brn3c:mGFP) line expresses EGFP in the plasma membrane, while hair cells in the myo6b:EGFP line express a cytosolic reporter (133, 134). Finally, behavioral assays provide a strong counterpoint to cellular assays. Zebrafish larvae show a dose-dependent reduction in rheotaxis (orientation to water flow) after exposure to neomycin or cisplatin (135, 136). Other groups have used startle response assays to measure lateral line damage (137, 138). While useful, behavioral assays should be interpreted with caution. Acoustically-driven startle responses are mediated by both the lateral line and inner ear, and bath application of ototoxins preferentially damages the lateral line; the drug generally does not rapidly penetrate the ear (137, 139). Therefore, an animal can have few lateral line hair cells and still show a robust acoustic startle response. Similarly, rheotaxis depends on multiple sensory systems, including vision and tactile responses (140). Conducting rheotaxis assays under far red light greatly reduces the visual component of the response but somatosensory cues are still present (135). Recently, transgenic fish lines engineered with a fluorescent calcium reporter have provided detail on hair cell function with single-cell resolution, allowing researchers to distinguish toxic effects on MET vs. synaptic transmission (141, 142). Physiological and behavioral techniques are labor-intensive and not suitable for high-throughput screening. However, they provide an excellent complement to cell-based assays once a putative ototoxin is identified.

Several studies have used the lateral line to screen drug libraries for novel ototoxins. Ton and Parng (126) provided proof-of-concept for this approach by demonstrating that lateral line hair cells could be damaged by both known ototoxins such as gentamicin and by suspected ototoxins, including the chemotherapy agent vinblastine sulfate. Expanding on this concept, Chui et al. interrogated a library of over 1,000 FDA-approved drugs and known bioactive compounds and identified 95 potential hair cell toxins (12). Of these initial “hits,” 21 compounds were verified on retest, including antiprotozoals such as pentamidine and the anticholinergic compound propantheline. Both compounds damaged lateral line hair cells in a dose-dependent manner (12). Further, both drugs also damaged hair cells in utricular explants from the adult mouse, demonstrating concordant toxicity between the lateral line and mammalian inner ear. A smaller screen of 88 antineoplastic agents by Hirose et al. detected 13 hair cell toxins (143). Some compounds, such as the microtubule inhibitor vinorelbine and the tyrosine kinase imatinib, were already considered suspected ototoxins based on scattered case reports (144–147), but the zebrafish study was the first to confirm hair cell cytotoxicity with either drug. The anti-cancer screen also identified novel putative ototoxins, including sunitinib, another tyrosine inhibitor. In 2020, a targeted study by Davis et al. showed that CQ and HCQ damaged lateral line hair cells and observed similar damage in mouse cochlear cultures (51). This study provides further evidence for the suspected ototoxicity of these compounds—a particular concern given their use in COVID-19 patients as we previously described. In COVID-19 trials, CQ/HCQ has been given in combination with azithromycin, another potential ototoxin, but it's unclear the degree to which these drugs interact to cause hair cell toxicity. Rapid ototoxicity models offer excellent benefits for time-sensitive investigations and allow for studies of drug–drug interactions.

In addition to FDA-approved drugs, unregulated natural products also represent a potential cause of unrecognized hearing loss. Natural products come from a diverse array of sources, are accessible over the counter, and are often used as chemical scaffolds for drug development. Neveux et al. screened a library of 502 natural products in 2017 and identified nine putative ototoxins, including kaempferol and quercetin; two of the major components of the popular supplement Gingko biloba (131). Pharmacologic inhibition of the hair cell MET channel attenuated hair cell damage, suggesting that like aminoglycoside antibiotics, these plant flavonols entered hair cells in a transduction-dependent manner (105). However, ototoxicity of kaempferol and quercetin has yet to be confirmed *in vivo* in mammals.

As discussed above, genetic polymorphisms play a role in susceptibility to aminoglycoside ototoxicity. It is likely that the same polymorphisms, or those not yet identified, modulate ototoxicity by other drugs such as COVID-19 therapeutics. The zebrafish lateral line was previously used for a large-scale screen to identify novel genetic regulators of aminoglycoside-induced hair cell damage (9). This study demonstrated that mutations in several cilia-associated genes attenuate neomycin-induced hair cell damage, including genes associated with intraflagellar transport (9, 148). Follow-up studies demonstrated that mutations in the chloride/bicarbonate exchanger *slc4a1b* and the transcription factor *gcm2* also attenuate lateral line hair cell damage from both aminoglycosides and cisplatin (128, 149). Similar screens are necessary to identify genetic modulators of ototoxic responses to other drugs, particularly CQ/HCQ, azithromycin, and other COVID-19 therapeutics with known or suspected ototoxic potential.

### Computational Modeling

Computational models are an increasingly powerful tool to predict drug toxicity in an unbiased manner and are commonly used to predict cardio- or hepato-toxicity (14, 150–152). These models use molecular structure, physiochemical properties, and other drug fingerprints to compare a novel compound or suite of compounds to a database of known toxins and non-toxins [e.g., (14, 15, 153)]. The models then return an estimate of toxicity for further validation *in vitro* or *in vivo*. Models increasingly employ machine learning algorithms such as neural networks, Bayesian, and deep learning approaches and regularly achieve 70–90% accuracy (14, 154, 155).

Most cardiac toxicity models focus on identifying drugs that interact with a single target; the human ether-a-go-go related gene (*hERG*), which encodes a voltage-gated potassium channel critical for cardiac function (15, 153, 156). Having a single target simplifies the model: what is the likelihood that the novel structure in question can serve as a ligand for hERG? By contrast, hepatoxicity comprises a heterogenous set of compound classes including protein kinase inhibitors, herbal supplements, and anti-cancer agents (157–159). Models of drug-induced liver injury predict phenotypic toxicity rather than specific ligand binding and therefore resemble ototoxicity in terms of the complexity needed for predictive power.

Despite the power of *in silico* approaches to identify emerging toxins, we know of only two published studies that have developed algorithms for ototoxicity (17, 18). Both used a Bayesian approach and trained their models on a dataset of ototoxins and non-ototoxins. They then tested their models using a test set composed of a subset of their original training set, achieving accuracies of 85–88%. However, both studies used flawed data to train their model, as their training dataset of *non-ototoxic* compounds contained drugs for which ototoxicity is reported in the literature. For example, both studies included the chemotherapy agents gallium nitrate and lapatinib in their dataset of ear-safe compounds, despite clinical reports of ototoxicity (160) or demonstrated hair cell toxicity in animal models (161). Flawed training datasets will yield less accurate predictions of ototoxicity when applied to new chemical structures. Published models should also use *in vitro* or *in vivo* assays to validate predicted toxins, and proposed models should be applied to new compounds not in the training dataset. These approaches are important steps to address the need for rapid, accurate *in silico* identification of emerging ototoxins. *In silico* approaches are defined by their use of computer models. Here, *in silico* screening involves the use of chemical structures to identify a subset of compounds with potentially similar structure-activity relationships, i.e., ototoxicity, that can then be tested *in vitro* or *in vivo*.

We have adapted published computational toxicity models to identify ototoxins based on chemical structure. Here, we briefly describe one such model to illustrate the validity of a robust computational approach to ototoxicity prediction. A complete description of the model is beyond the scope of this review and will be published elsewhere. In this example, our model shares features with published ototoxicity models but we use a smaller but well-validated training dataset with strict classification of ototoxic vs. non-ototoxic drugs based on peer-reviewed literature drawn from PubMed. As input, we use isomorphic SMILES (simplified molecular-input line-entry system) data for each drug derived from PubChem; this format uses line notation to describe chemical structure as a series of fingerprints and is commonly used for toxicology modeling (151, 159, 162). This model uses a binary classifier approach where positive scores represent ototoxicity and negative scores represent non-ototoxicity. Predictions are based on summing a weighted Tanimoto Similarity Index (*T*) for the given test compound (*c*) across each compound in the training set (database of known ototoxins, *o*, and non-ototoxins, *n*). Known ototoxin and non-ototoxin comparison scores are summed as separate values and multiplied by separate weights as seen in Equation (1). This approach allows us to analyze a test compound's ototoxic and non-ototoxic potential independently, improving prediction accuracy in our preliminary studies. Tanimoto-based approaches have been used for similar models of liver toxicity (152).

(1) $$\begin{array}{r} Score_{c}=\sum \alpha_{o}T_{o,c}+\sum \beta_{n}T_{n,c} \end{array}$$

**Figure 2:**
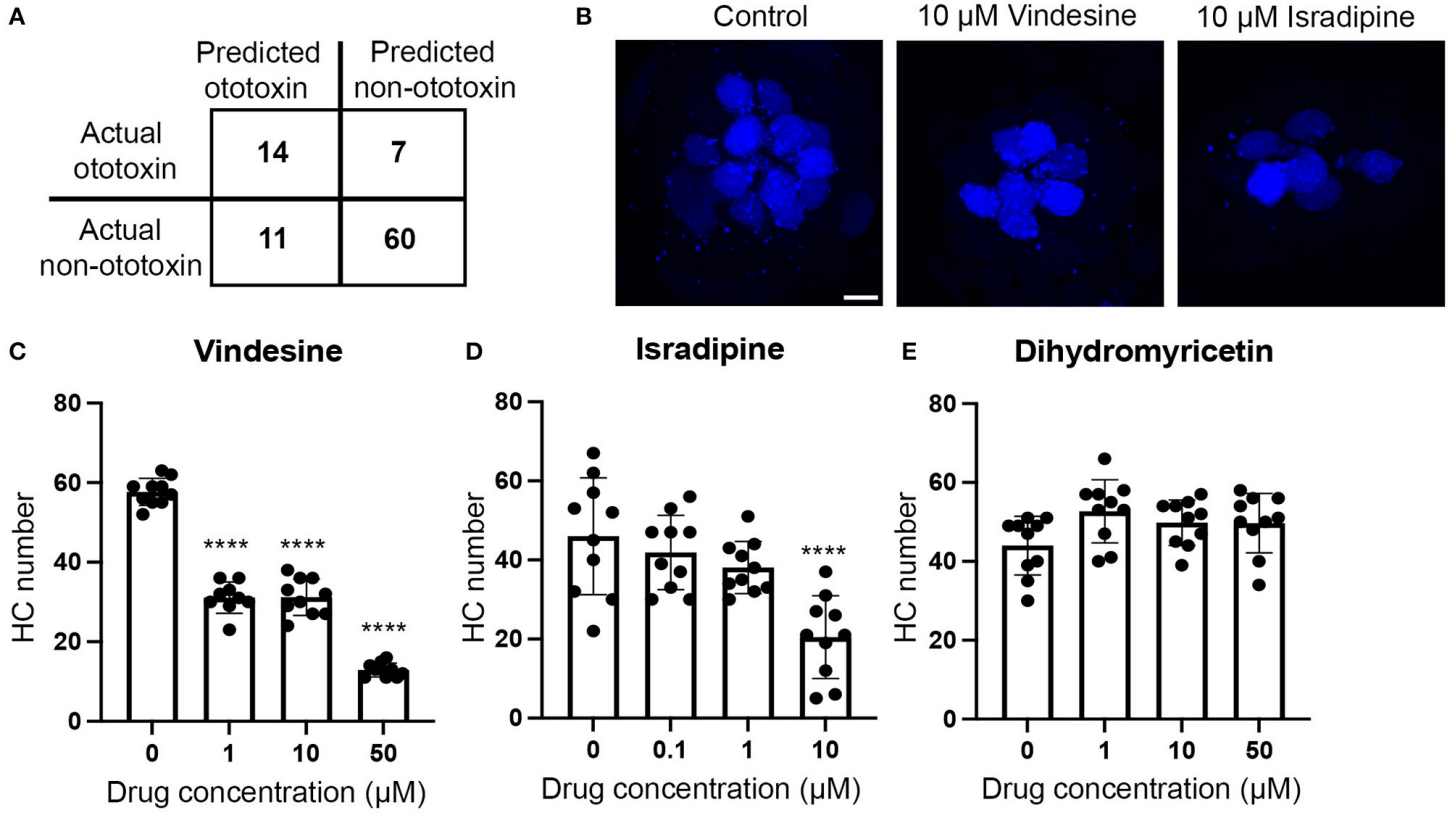
Illustration of how computational and experimental approaches can be combined to identify new ototoxins. **(A)** Confusion matrix output demonstrating accuracy of our Tanimoto-based algorithm. We used 70% of our ototoxin database to train the model and 30% of our database as probe structures to test accuracy. The model correctly predicted 14 of 21 ototoxins (66%) in the probe test set and 60 of 71 non-ototoxins (84%) for an average accuracy of 75%. **(B–E)** We then used our model to predict ototoxicity from the PubChem database of ~10,000 ototoxins and validated predicted ototoxins *in vivo* in the zebrafish lateral line. **(B)** Vindesine and isradipine are toxic to lateral line hair cells. Confocal images show the M2 neuromast from fish treated for 24 h with DMSO (vehicle control, left), 10 μM vindesine (middle), or 10 μM isradipine (right). Hair cells were live-labeled with DAPI. The scale bar on the left panel = 5 μm and applies to all images. **(C–E)** Quantification of hair cell numbers in five head neuromasts per fish. Numbers were summed to arrive at one value per animal. **(C)** Vindesine significantly damages hair cells at all concentrations tested [One-way ANOVA, *F*_(3, 35)_ = 268.4, *p* < 0.0001]. **(D)** Isradipine also caused significant hair cell loss [*F*_(3, 36)_ = 10.97, *p* < 0.0001]. 50 μM isradipine was toxic to fish (data not shown). **(E)** Dihydromyricetin did not cause hair cell loss [*F*_(3, 36)_ = 2.42, *p* = 0.07]. Bonferroni-corrected *post-hoc* tests indicate significant differences from controls ****p < 0.001. *N* = 9–10 animals per group, data are presented as mean + s.d. and dots represent individual animals.

Our model is 75% accurate at predicting ototoxic vs. non-ototoxic structures. Accuracy was based on application of a confusion matrix that used 70% of our dataset to train the model and the remaining 30% as probe structures to classify ototoxins through the application of Equation (1) (Figure 2A). In this example, the model identified more predicted non-ototoxins then ototoxins, consistent with the over-representation of non-ototoxins in the training dataset. As proof of concept, we used our model to query a PubChem database of ~10,000 drugs to determine the model's ability to identify new ototoxins. Of the 180 predicted ototoxins, some, such as kanamycin, were known ototoxins represented in our training dataset, while other predicted ototoxins were not previously reported in the literature. We then tested three putative ototoxins in the zebrafish lateral line to validate hair cell toxicity using vital dye labeling methods described above for lateral line studies (Figures 2B–E). The chemotherapy agent vindesine and the anti-hypertensive isradipine both killed hair cells, while the antioxidant dihydromyricetin did not cause damage, even at concentrations five times higher than ototoxic doses of vindesine or isradipine (Figure 2E). Collectively, Figure 2 illustrates how computational and experimental approaches can be combined to identify new ototoxins. This strategy should be highly useful when applied to databases of COVID-19 therapeutics, allowing researchers to rapidly identify potential ototoxins based on chemical structure, then opportunities to validate these ototoxins in auditory cell lines or the lateral line system.

Future ototoxicity modeling efforts will benefit from deep learning approaches such as convolution neural networks. Unfortunately, these algorithms traditionally rely on large datasets. Recent advances in few-shot learning and data augmentation methods allow for deep learning approaches using small datasets—an excellent fit for the relative paucity of validated ototoxins in our training dataset (163–165). Another option is to use transcriptomic data for model fitting, either in lieu of or in addition to molecular fingerprints. For example, in 2019, Wang et al. used gene expression signatures of drug-induced liver injury as input data to train a deep neural network to predict hepatotoxicity based on transcriptomics profiles (166). Multiple studies have characterized the hair cell transcriptome after exposure to aminoglycosides and cisplatin (167–170). These gene expression profiles could provide input data to train ototoxicity prediction models based on transcriptome signatures for novel compounds. Finally, future models could be trained to identify drug–drug interactions that increase ototoxic potential; an effort that is sorely needed to address the many medications administered to COVID-19 patients.

## Conclusion

Over 100 drugs are known or suspected ototoxins and hundreds of new candidate otoprotectants are developed in research labs each year. Therefore, we argue that ototoxicity screening should be a required step in the drug development process. Use of rapid models such as auditory cell lines, larval zebrafish, and computational models make it relatively easy for researchers to screen lead compound libraries for ototoxicity and to select non-ototoxic leads for further preclinical development. Such screening can be done in-house by larger organizations; and given the number of labs that specialize in these high-throughput models, there are ripe opportunities for collaboration between drug development groups and ototoxicity researchers.

As of June 2021, there were more than 3,000 trials testing clinical interventions on over 900 drugs. It would require a Herculean effort for any one group to initiate audiometric studies at this scale—a consortium approach is required. Further, given the hundreds of drugs and their various dosing regimens in clinical trials, it is crucial to begin audiometric screening. Although we have spoken to several experts in human ototoxicity monitoring in the U.S. and Europe, none were aware of clinical trials collecting audiometric data, as called for by multiple researchers (45, 49, 171, 172). Preclinical screening at *in silico, in vitro*, and *in vivo* levels as described above can provide an earlier indication of the potential for ototoxicity and identify the subset of candidate therapeutics for treating COVID-19 that need to be monitored for ototoxicity as for other widely-used clinical therapeutics, like aminoglycosides and cisplatin (100, 173, 174).

The model systems we describe have been used to identify and characterize new ototoxins, probe mechanisms of damage, and to rapidly identify novel protectants. Otoprotectant identification stems from the same characteristics that make these models so valuable for ototoxin studies because researchers can screen large numbers of putative protective compounds. To date, most of the otoprotection work has focused on an important but limited number of known ototoxins, particularly aminoglycoside antibiotics and platinum-based chemotherapy agents. When it comes to emerging ototoxins, particularly those in consideration as COVID-19 therapeutics, there is little work on the otoprotection side of the equation; an area ripe for exploration using rapid *in vitro, in vivo*, and *in silico* models.

## Author Contributions

AC, RB, CT, and PS wrote sections of the manuscript. AC, RB, JH, CT, and PS reviewed and edited the manuscript. RB, AC, and JH contributed the data for Figure 2. All authors contributed to the article and approved the submitted version.

## Conflict of Interest

RB was employed by the company Rewire Neuroscience. The remaining authors declare that the research was conducted in the absence of any commercial or financial relationships that could be construed as a potential conflict of interest.

## Publisher's Note

All claims expressed in this article are solely those of the authors and do not necessarily represent those of their affiliated organizations, or those of the publisher, the editors and the reviewers. Any product that may be evaluated in this article, or claim that may be made by its manufacturer, is not guaranteed or endorsed by the publisher.
